# Spontaneous Relocation of a Posterior Dislocation of Mobile Bearing in a Medial Unicompartmental Knee Replacement

**DOI:** 10.1155/2012/230430

**Published:** 2012-05-31

**Authors:** Hussein Noureddine, Jaimes Aird, Paul Latimer

**Affiliations:** ^1^Department of Orthopaedics, Morriston Hospital, Abertawe Bro Morgannwg University Health Board, Morriston, Swansea SA6 6LZ, UK; ^2^Orthopaedics Department, Frenchay Hospital, North Bristol NHS Trust, Frenchay Park Road, Bristol BS16 1LE, UK; ^3^Department of Orthopaedics, Yeovil District Hospital NHS, Trust, Higher Kingston, Yeovil BA21 4AT, UK

## Abstract

We describe a case of spontaneous relocation of a posterior dislocation of the mobile bearing in a medial unicompartmental knee replacement, prior to surgical intervention. We are unaware of any similar cases in the published literature. This paper highlights some clinical issues around this type of dislocation.

## 1. Case Report

A 58-year-old female presented to the Accident and Emergency Department with severe pain in her right knee and unable to weight bear, after sustaining trauma with a valgus force to the knee in a flexed position. An Oxford mobile bearing medial unicompartmental knee arthroplasty had been performed 3 years previously for severe right-knee medial compartment osteoarthritis, with a correctable varus deformity of 10 degrees. She had a 6 mm meniscal bearing inserted at the time of surgery, and no medial collateral ligament release was performed. The knee was felt to be well balanced, and the meniscal bearing was stable. After the surgery she had an uneventful recovery and returned to full pain-free function.

On examination she had a painful nonswollen knee, with range of movement limited from thirty to sixty degrees, and tenderness over the medial joint line. There was significant medial-lateral laxity. The knee felt unstable, but medial collateral ligament felt intact. Distal neurovascular status was intact.

**Figure 1:**
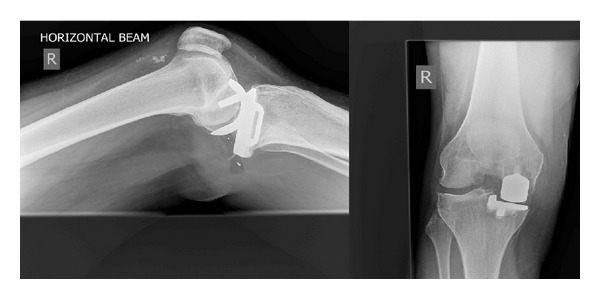
Lateral view and AP view radiographs of the knee taken upon presentation at the Accident and Emergency Department.

 Radiographs were performed and showed a posterior dislocation of the meniscus (Figure 1), and it was decided to proceed to theatre for a closed/open reduction of the dislocated bearing.

**Figure 2:**
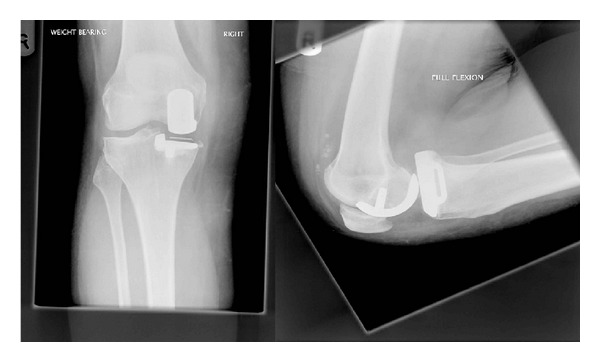
AP view and lateral view radiographs of the knee prior to replacing the mobile bearing.

**Figure 3:**
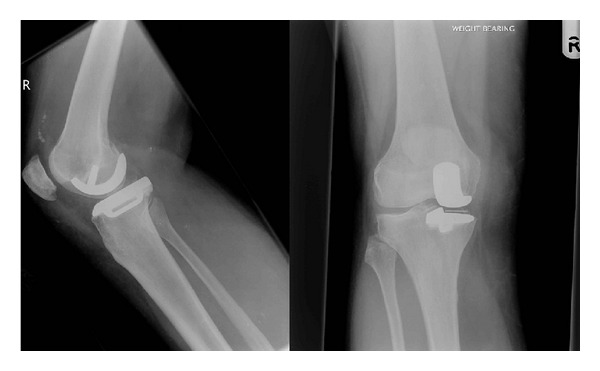
Lateral view and AP view radiograph of the knee taken following operation.

On examination under anaesthetic, the knee was found to have a full range of movement and to be stable prior to any intervention. Spontaneous relocation was considered (Figure 2), but due to concerns regarding recurrent dislocation and possible damage to the bearing, a mini-open approach to the knee was made, and the bearing was observed to have relocated. However, it had significant wear on its superior articular surface in the form of pitting and a small area of delamination of about five squared millimetres, and it was replaced by a 7 mm polyethylene liner (Figure 3). Postoperatively she made a good recovery and 6 months postoperatively has had no further trouble. 

## 2. Discussion

Unicompartmental knees were first developed in the 1950s by McKeever and MacIntosh. In 1978 the meniscal bearing was introduced by Goodfellow. The prosthesis has undergone a series of evolutionary steps to give us the mobile and fixed designs in use today. In the recent literature, there has been controversy as to whether mobile bearings provide superior results in the long term, over fixed bearing implants by virtue of reduction in wear and improved kinematics [1–3]. Potential problems with UKR are that they are technically demanding procedures, which have been shown to have a higher revision rate than TKR [4, 5]. The mobile bearings may also dislocate, and rates have been quoted as being between 0.5% and 10% [6–10]. These dislocations tend to be anterior or lateral and have been found to occur more frequently in the first year postoperatively. There have been reports of these being reduced with both closed and open techniques [4].

Posterior dislocation of the meniscal bearing poses additional diagnostic and treatment issues, in comparison to anterior dislocation. The soft tissue structures at the back of knee lead to the potential for significant neurovascular compromise as a result of this type of dislocation. Moreover, the soft tissue coverage makes diagnostic examination and closed reduction more challenging. All this, in addition to the lack of evidence, persuaded us to adopt a cautious approach and insert a larger bearing. In this case, the bearing reduced spontaneously prior to surgical intervention. Nevertheless we opted to open the joint both to confirm congruent reduction and to inspect the prosthesis for damage. By exchanging the polyethylene meniscus but retaining the metal components, our patient has had no further functional problem at a followup of two years.

No benefits or funds were received in support of the study.

Patient's written consent to report the case was obtained.
